# Arts engagement supports social connectedness in adulthood: findings from the HEartS Survey

**DOI:** 10.1186/s12889-021-11233-6

**Published:** 2021-06-24

**Authors:** Rosie Perkins, Adele Mason-Bertrand, Urszula Tymoszuk, Neta Spiro, Kate Gee, Aaron Williamon

**Affiliations:** 1 Centre for Performance Science, Royal College of Music, London, UK; 2 Faculty of Medicine, Imperial College London, London, UK

**Keywords:** Arts, Loneliness, Qualitative, Social connectedness, Social wellbeing, Survey

## Abstract

**Background:**

Loneliness is a public health challenge, associated with premature mortality and poorer health outcomes. Social connections can mitigate against loneliness, and there is evidence that the arts can support social connectedness. However, existing research on the arts and social connectedness is limited by focus on particular age groups and arts activities, as well as a reliance on typically small-scale studies.

**Methods:**

This study reports survey data from 5892 adults in the United Kingdom, closely matched to the national profile in terms of sociodemographic and economic characteristics. It investigates the extent to which arts engagement is perceived to be linked with feelings of social connectedness, which forms of arts engagement are reported as most connecting, and how. Data were collected via the *HEartS Survey*, a newly designed tool to capture arts engagement in the United Kingdom and its associations with social and mental health outcomes. Demographic and quantitative data, pertaining to the extent to which arts engagement is perceived to be linked with social connectedness, were analysed descriptively. Qualitative data pertaining to respondents’ perceptions of how arts engagement is linked with feelings of social connectedness were analysed using inductive thematic analysis.

**Results:**

Results demonstrated that the majority of respondents (82%) perceive their arts engagement to be linked with feelings of social connectedness at least some of the time. The forms of arts engagement most linked with feelings of social connectedness were attending a live music performance, watching a live theatre performance, and watching a film or drama at the cinema or other venue. Four overarching themes characterise how arts engagement is perceived to facilitate feelings of social connectedness: social opportunities, sharing, commonality and belonging, and collective understanding.

**Conclusions:**

The findings suggest that arts engagement can support social connectedness among adults in the UK through multiple pathways, providing large-scale evidence of the important role that the arts can play in supporting social public health.

**Supplementary Information:**

The online version contains supplementary material available at 10.1186/s12889-021-11233-6.

## Background

Loneliness is an unpleasant emotional experience of dissatisfaction with the quality or quantity of one’s social relationships [1]. Distinct from social isolation per se, loneliness can be emotional (the absence of close, emotional connections with others), social (the absence of social relationships or contact) [2], and existential (feeling disconnected from others and the universe) [3]. Loneliness is a public health problem, associated with a 26% increase in the risk of premature mortality [4, 5] as well as poorer cardiovascular and mental health outcomes [6]. Depression has been associated with increased risk of loneliness in young, middle-aged, and older adults, with those who report depression also reporting high levels of loneliness at around 38% [7]. Other mental health disorders such as obsessive-compulsive disorder and phobia are also linked with loneliness [8]. As well as prolonged mental illness, loneliness is associated with ethnic minority status and living alone, with substance use problems [9], as well as socio-demographic and health-related factors within specific age groups, particularly younger adults (under 25 years) and older adults (over 65 years) [7, 10].

Given this context, attention has turned to ways to reduce loneliness. Recent syntheses of intervention studies for loneliness evidence a diverse range of intervention types, but with no effects seen in controlled studies [1, 11]. Importantly, the reviews revealed that loneliness is not often a primary outcome measure but, rather, is typically reported along with other, related social measures. Indeed, loneliness is closely linked as a concept with (lack of) social connections, and these two terms are often used interchangeably in the literature. Conceptualised by Lee and Robbins, social connectedness refers to how one thinks of oneself in relation to other people, including how emotionally distant or connected one feels to individuals and society [12]. It can be defined as ‘the opposite of loneliness, a subjective evaluation of the extent to which one has meaningful, close, and constructive relationships with others (i.e., individuals, groups, and society)’, including caring about others, feeling cared for, and belonging to a group [13] . Moreover, social connectedness provides a useful counter concept to the deficit notion of loneliness [14], placing attention on what people *can* do in order to build social connections that are meaningful and fulfilling. Others explicitly position social connectedness as a potential means of reducing loneliness [15].

In this respect, the arts have emerged as an area of practice with potential for fostering social connectedness and reducing loneliness. Review articles, while pointing out limitations in the evidence base such as inconsistency in measurement tools and reporting of results [16], suggest that engagement in the arts can particularly support older adults. Dadswell and colleagues, for example, report a conceptual review indicating that participatory arts can strengthen and build relationships through social interaction, tackle inconsistences between expected and real relationships through enhancing self-worth and feelings of belonging, and allow older adults to contribute inclusively to their community [17]. Similarly, a review of forty-four studies indicated that participatory arts can promote reciprocal relationships among older adults, care givers, and the wider community [18]. Others concur that community-engaged art programmes are a ‘promising tool’ for addressing loneliness in older adults [19] and point to the connecting benefits of indoor gardening and visual art discussions [20].

Aside from review articles, which tend to focus on older adults, there is also a large body of work reporting that specific arts and cultural activities and interventions reduce feelings of loneliness and/or enhance social connections. Work with museums, for example, has shown that art and reminiscence workshops can support social inclusion and community building [21] and that ten-week museum programmes can help to reduce isolation and loneliness through creating opportunities for social inclusion, social engagement, and enhanced communication [22]. Furthermore, frequent engagement with museums, galleries, and exhibitions may be a protective factor against loneliness in older adults [23], and art therapy in art museums can promote social connectedness in older adults [24]. In music, a significant effect of music therapy on loneliness was shown among institutionalized older adults with mild depression compared with controls [25]. Drumming has been reported to enhance feelings of togetherness, belonging, and connectedness among soldiers experiencing PTSD [26] and mental health service users and their carers [27], while group singing has been shown to enhance emotional closeness between mothers and babies [28], and to mediate swift social bonding [29]. Sustained choral singing is also shown to enhance social connectedness [30] and reduce loneliness [31], and private music engagement has been shown to provide a sense of social connection and social surrogacy [32], or in other words people ‘resort [ing] to temporary substitutes, so-called social surrogates, if direct social interaction is not possible’ [33] . Finally, university students listen to music to reduce loneliness [34], a finding also seen in older adults [35].

In other art forms, older adults who read for pleasure were found to be significantly less lonely than adults who did not [36]. Indeed, arts activities including reading books were found to be successful predictors for social connectedness in older people [37]. In dance, line dancing in women aged over 60 years was found to increase social activity, including further community involvement and charitable work [38], and dancing in synchrony has been linked with enhanced social bonding [39]. Textile crafting has also been linked with social interaction and connectedness [40] as well as social connectedness through belonging [41]. In addition, community arts programmes, including crafts activities such as painting, pottery, and printmaking, were found to be particularly effective in mitigating loneliness among older adults and producing social inclusion [42]. Similarly, sandplay therapy was shown to reduce levels of loneliness and build social networks among migrant women [43]. Further to this, arts activities provided by social enterprises directly counteracted social isolation and loneliness in rural communities and enabled users to build social connections [44]. Finally, the arts can support community development through building group solidarity as well as individual and group identities [45] and can be used within community regeneration [46].

Although many studies have focused on older adults, there is also a body of research investigating how music can contribute towards positive social wellbeing in young adults. For example, musical engagement can help young people to strengthen relationships through the sharing of songs, and live music events can establish feelings of social connections between young adults and their friends and family [47]. Similarly, music can promote feelings of social connectedness, with young people viewing music as a means of connecting, sharing thoughts, opinions, and passions within others [48]. Music has also been identified as playing a key role in building shared identities amongst young adults. North and Hargreaves demonstrated how music can act as a ‘badge’ through which young people can judge others and convey aspects about themselves, with members of musical subcultures being found to hold positive perceptions of those with similar musical tastes [49]. Others have similarly highlighted how music acts as an important tool for the construction of group identities, with shared musical tastes creating a sense of belonging and community amongst young adults and music acting as a means of forging connections with others [50]. In addition, music making has been shown to support the social inclusion of young people within the criminal justice system, providing a space where participants could find respite from stigma and enjoy learning as part of a group [51].

While the majority of studies on young adults and the arts have focused on music, wider arts activities have also been shown to have positive social benefits for this group. Interviews and focus groups with young adults have demonstrated that attending recreational dance classes can establish as a sense of connection to cultural heritage and community, and develop social relationships [52]. Dance has been framed as an engaging social activity, with young women who danced regularly reporting higher rates of mindfulness and life satisfaction [53]. Furthermore, dancing was found to contribute towards the social wellbeing of young adults with autism, reducing tension and anxiety whilst building empathy, social skills, and ability to cope [54]. Participation in theatre groups has also been shown to promote social wellbeing, providing opportunities for self-expression, problem solving, and life improvement amongst vulnerable young adults that developed collective efficacy [55].

These studies illuminate several gaps in current understanding of the relationships between the arts and social connectedness. First, review articles focus predominantly on older adults, meaning that synthesised or large-scale knowledge for other age groups is largely lacking. Second, a majority of the studies investigating the social wellbeing of young adults have focused on music activities or arts interventions undertaken with targeted groups. Within much of the existing literature, certain arts activities (including museum/gallery attendance and music) are prioritised, meaning that other forms of arts engagement – particularly those conducted at home, or in less structured ways – can be overlooked. Moreover, the majority of studies are relatively small-scale, and – while there is consensus that arts engagement can support social connectedness – there is a lack of scrutiny of the psychosocial processes through which this happens. This article sets out to meet these gaps, answering three research questions:

- RQ1: To what extent do adults perceive a link between their arts engagement and feelings of social connectedness?
- RQ2: Which forms of arts engagement are most reported as linked with feelings of social connectedness?
- RQ3: How is arts engagement perceived to facilitate feelings of social connectedness?

## Methods

### Method

Data were collected via the *HEartS Survey* (Health, Economic, and Social impacts of the ARTs), which was designed to capture current arts engagement in the United Kingdom (UK) and to explore its sociodemographic characteristics and correlations with mental and social wellbeing [56]. The *HEartS Survey* consists of seven sections: (1) demographics, (2) reported frequency and nature of arts and cultural activities, (3) open questions on arts engagement and social connectedness, (4) mental health and wellbeing, (5) physical activity, (6) social wellbeing, and (7) household income and arts spending [56]. This study draws on data from sections 1, 3, 4, 6, and 7.

Demographic information was collected in sections 1 and 7 regarding age, gender, geographic region, ethnicity, educational qualifications, living situation, and household income. Validated scales were used to collect information about social connectedness, loneliness, and depression in sections 4 and 6. Social connectedness was measured using the 15-item Social Connectedness Scale [57], where a higher score (on a scale of 0–75) indicates more connectedness to others. Loneliness was measured using the Three-Item Loneliness Scale [58, 59], which identifies those scoring 6 or higher out of a possible 9 as lonely. Depression was measured using the eight-item Center for Epidemiologic Studies Depression Scale (CES-D). The number of selected depressive symptoms is summed, generating a score (0 to 8). A score of three symptoms or more (out of the possible eight) has been commonly used for identifying cases of depression [60]. Where possible, we used standardised Office for National Statistics Census questions (including geographic regions, ethnicities, educational qualifications, and living situations) and attempted to match our sample to the national profile. Further information on the validated measures and the HEartS Survey is available elsewhere [56, 61].

Data to answer the research questions were drawn from three questions in section 3: *Q1. Does your engagement with the arts and cultural activities you have told us about today [*i.e.*, those identified by each respondent in section 2] help you to feel connected with other people?* (measured on a 5-point scale: not at all, a little, around half the time, often, always); Q2. *Of the arts and cultural activities you have told us about, which makes you feel most connected to other people? (Please select just one activity)* (drop-down choice of all arts activities reported by the respondent in section 2); Q3. *Why does this activity make you feel connected to other people? Please write in as much detail as possible and include examples or stories where appropriate* (open response).

Ethical approval for the research was granted by the Conservatoires UK Research Ethics Committee (CUK REC) on 16 February 2019. All methods were performed in accordance with CUK REC and British Psychological Society (BPS) guidelines and regulations, and respondents gave informed consent to participate. Links to support (e.g., for mental health) were provided at the end of the survey.

### Respondents

UK-based respondents were recruited to complete the *HEartS Survey* through an online data collection platform, Qualtrics, over a period of 6 months between March and August 2019. Data collection quotas were set for gender, age, geographical region, ethnicity, and education following the overall distributions of these key sociodemographic variables in the UK 2011 Census [56].

A total of 11861 respondents started the survey. Of these, 1623 did not consent to participate in the survey and stopped at the consenting process. A further 3219 respondents were excluded after answering initial sociodemographic questions as the quotas for their characteristics were already reached. A further 969 were excluded due to completing the survey in under four minutes (i.e., speeding through the survey, *n* = 97) or providing nonsense or abusive responses to open questions (*n* = 872) [56]. Of the remaining 6050 participants, 5892 answered Q1 above and therefore constitute the sample for this study. All respondents who completed the survey were paid a modest fee via the Qualtrics platform.

Full sociodemographic characteristics are detailed in Supplementary Table 1. Respondents were adults living in the UK aged 18–25 (13%), 26–35 (21%), 36–45 (17%), 46–55 (15%), 56–65 (17%), 66–75 (14%), and 76–94 (2%) years. 51% of respondents identified as women. 89% identified as White British or Irish, or other White background, 5% as Asian ethnic backgrounds, 2% as Black ethnic backgrounds, 2% as Mixed ethnic backgrounds, and 1% as any other ethnic background. Respondents lived across regions in the UK, with the largest group (27%) living in London or the South East. 4% had no formal educational qualifications, 12% other vocational and foreign qualifications, 26% GCSE, O Level, AS level, and NVQ Level 1–2 qualifications, 23% A level, baccalaureate, and NVQ Level 3 qualifications, and 36% university degree and NVQ Level 4–5 qualifications. 20% of respondents lived alone, 35% with a partner only, 29% with children (with or without a partner), and 15% with family, house share, or other. The final sample matches the national profile closely in terms of sociodemographic and economic characteristics, but both the rates of loneliness (46%) and depression (78%) in this sample are higher than those in the general population. As discussed elsewhere, this may be connected to the nature of the non-probability, online survey [56].

### Analysis

Descriptive statistics using quantitative data for RQ1 and RQ2 are reported below. Further analyses from the *HEartS Survey* are documented elsewhere [56]. Analysis of qualitative data for RQ3 (excluding those who answered ‘no’ to whether the arts connected them to others or provided nonsense responses, leaving *n* = 4352) was conducted in NVivo, following an inductive thematic analysis that proceeded in five-steps. First, 10% of the data for each art form selected in Q2 were analysed by one author (AMB) to identify codes salient to RQ3. This analysis was inductive and cross-checked by another author (RP). Second, the agreed coding scheme was applied to another 40% of the data by AMB, with codes grouped into suggested sub-themes and themes. The coding and the grouping were cross-checked by RP, resulting in reassignment of some codes and relabelling or regrouping of some of the sub-themes and themes. Third, the revised coding scheme was applied to the remaining 50% of the data by AMB. Fourth, the pre-final coding and groupings were cross-checked by RP before RP and AMB agreed the final set. Finally, this final set were cross-checked by a third author (UT).

## Results

### To what extent do adults perceive a link between their arts engagement and feelings of social connectedness? (Q1)

Of the sample of 5892, 4785 (82%) reported that arts engagement made them feel socially connected, as summarised in Table 1. 35% reported that this occurred a little, 17% around half of the time, 22% often, and 8% always.

**Table 1:** The extent to which adults in the UK perceive a link between their arts engagement and feelings of social connectedness

*Does your engagement with the arts and cultural activities you have told us about today help you to feel connected with other people?*	*Number*	*Percent*
not at all	1107	19
a little	2039	35
around half of the time	997	17
often	1289	22
always	460	8

### Which forms of arts engagement are most reported as linked with feelings of social connectedness? (Q2)

Table 2 summarises the arts and cultural activities identified by respondents as most linked with feelings of social connectedness. The top three activities identified were attending a live music performance (*n* = 1265, 26%), watching a live theatre performance (*n* = 465, 10%), and watching a film or drama at the cinema or other venue (*n* = 455, 10%).

**Table 2:** Forms of arts engagement most linked with feelings of social connectedness

*Of the arts and cultural activities you have told us about, which makes you feel most connected to other people?*	*Number*	*Percent*
Been to a live musical performance	1265	26
Watched a theatre performance live	465	10
Watched a film or drama at a cinema or other venue	455	10
Read as a past-time activity	336	7
Been to an exhibition, museum, or collection of art, photography, sculpture/other arts	267	6
Played a musical instrument or sang	262	6
Deliberately listened to recorded music	226	5
Listened to audio books or podcasts	198	4
Attended a book club where people meet to discuss and share books	170	4
Been to an event connected with books or reading	146	3
Done any form of crafts	144	3
Been to street art, public art displays, admired architecture or an historical monument	134	3
Done photography, film, video making, or other related pursuits as a past-time activity	132	3
Written as a past-time activity	101	2
Practised, rehearsed, or performed dance as a past-time activity	96	2
Done painting, drawing, printmaking, sculpture, or other related pursuits	82	2
Written or created music	74	2
Practised, rehearsed, or performed a play, drama, opera, musical theatre, circus skills	65	1
Watched a dance performance live	63	1
Been to a convention, show, or fair relating to crafts or decorative arts	63	1
Other	41	1

### How is arts engagement perceived to facilitate feelings of social connectedness? (Q3)

The qualitative analysis revealed 4687 codes organised into four key themes, comprised of 12 sub-themes and, where required, five sub-sub-themes (see Table 3). For each sub-theme, the evidence is discussed with indicative quotations selected from across the range of art forms. Supplementary Table 2 presents the number of citations across each form of arts engagement for each sub-theme and sub-sub-theme, supported by indicative evidence.

**Table 3:** Summary of themes for how arts engagement is perceived to facilitate feelings of social connectedness

*Themes and (sub)sub-themes*	*Description*
**1. Facilitating social opportunities**	**The arts facilitate social opportunities**
1.1 Meeting new people	The arts provide opportunities to meet new people and to make new friends
1.2 Socialising and interacting with others	The arts encourage and facilitate generalised social interactions
1.2.1 Conversing about art	The arts can act as a catalyst for conversations
1.2.2 Negating pressures of social interaction	The arts facilitate interaction and connection without pressures of social situations
**2. Facilitating sharing**	**The arts facilitate opportunities for shared experiences**
2.1 Sharing experiences	The arts act as a shared experience creating bonds between those involved
2.1.1 Sharing emotions	The arts allow people to share emotional responses and to express feelings
2.1.2 Sharing art with others	When artists share their art, it creates a sense of connection between them and their audience
2.2 Shared purpose	The arts provide people with a sense of shared purpose
**3. Facilitating commonality and belonging**	**The arts facilitate feelings of similarity and belonging**
3.1 Being part of something	The arts allow people to feel part of, and belonging to, something bigger than themselves
3.1.1 Enabling proximity to others	The arts elicit feelings of direct and indirect proximity with other people and times
3.2 Connecting through common interests	The arts connect people with common interests, specific to the arts activity
3.3 Connecting with likeminded people	The arts connect people with common tastes and opinions, regardless of the arts activity itself
**4. Facilitating collective understanding**	**The arts facilitate learning from and about other people**
4.1 Learning from and with others	The arts allow people to learn new skills and knowledge from and with others
4.2 Relating to others	The arts allow people to relate to others
4.3 Learning about others	The arts provide a means of learning about other people, places, and times
4.4 Connecting with different people and cultures	The arts expose people to different cultures and bring together people from different backgrounds
4.5 Connecting with different viewpoints	The arts expose people to different viewpoints and opinions

#### Theme 1: facilitating social opportunities

The first theme reflects how the arts facilitate social opportunities. Sub-theme 1.1 focuses on how the arts provide opportunities to meet new people:

It’s lovely to meet new people and mutually share perspectives [been to an exhibition, museum, or collection of art].

[Because] anyone can come there you meet a lot of nice and interesting people [street art, public art, architecture, historical monument].

It is sociable and a good way of meeting people [practised, rehearsed, or performed dance].

As well as meeting new people, the arts were also identified as a means of forging new friendships:

Once I went to a music festival and I ended up with many newfound friends as we had similar music tastes [live musical performance].

It’s actually how I met most of my friends. I was in the school library drawing when a group of four people with sketchbooks came up to me and started complimenting my work. We kept talking and now we’re all best friends [painting, drawing, printmaking].

I’ve been interested in photography since I was about five, when I played around with my Dad’s camera and took pictures of random things, myself and my parents. Ever since then I’ve been getting better cameras, getting more skilled at my interest and job, and have made lots of life-long friends due to it [photography, film, videomaking].

Linked with meeting new people and making friends, the arts were also reported to encourage and facilitate general social interactions (sub theme 1.2):

It brings everyone together as a social event. It’s a good opportunity to chat and build relationships with others [book or reading event].

We can appreciate the music together and we talk and hang out and generally have fun [live musical performance].

It’s a knitting group with a wide age range. The founder set it up as a group to help with mental health issues. It’s sociable, inspirational and makes what could be a solitary pastime into one to be shared [crafts].

In addition to general social interaction such as being with or talking with others, the arts were also identified as a specific catalyst for conservations (sub-sub-theme 1.2.1):

It gives me the opportunity to converse with other people, to exchange ideas and review conceptions [book club].

Admiring things with people and going to museums sparks conversation which helps me to connect [been to an exhibition, museum, or collection of art].

Street entertainment seems to create camaraderie between strangers, especially when it is unexpected and catches people by surprise. The public will often discuss what is going on with those around them, even if they have never met each other before [street art, public art, architecture, historical monument].

Finally, for a very small sub-set of the sample, the arts – particularly live music – facilitate interaction and/or connection while negating or mitigating some of the pressures of social interaction that they would otherwise experience (sub-sub-theme 1.2.2):

[I] get to enjoy the company of friends without having to worry about chatting and doing anything the whole time as you have something to watch [watched a theatre performance].

You can be together without having to engage with anyone and still share in the music and the feeling [live musical performance].

I am a mother of a large family. However, I consider myself a loner. Going to museums and such, when I am alone, makes me feel connected to the people who created the pieces. It’s akin to having a silent conversation with the artist. I enjoy this [exhibition, museum, or collection of art].

In sum, the first theme captures how the arts facilitate social opportunities including meeting new people and making new friends, encouraging general social interactions, catalysing conversations, and supporting feelings of connection for people who otherwise experience challenges with social interaction.

#### Theme 2: facilitating sharing

The second theme reflects how the arts facilitate opportunities for shared experiences. The first sub-theme concerns the sharing of experiences (sub-theme 2.1), with the arts acting as a shared experience that creates bonds between those involved:

The performance makes me feel more connected to people, like we’re sharing something [live musical performance].

It is the shared sense of witnessing something. It gives it a certain sense of permanence, and timelessness. Going to see something as evocative and inspiring as the reclining Buddha in Bangkok makes you think of all the other people that have stood where you are. It makes you contemplate how they felt. It gives you a far greater depth of perception and to my mind increases your human consciousness [street art, public art, architecture, historical monument].

Cinema is a shared experience despite being in the dark and not having contact with anybody [watch a film or drama at a cinema or other venue].

In addition to the more general sense of the arts being a shared experience, two sub-sub-themes further illuminate this idea. First, that the arts allow people to share emotions (sub-sub-theme 2.1.1):

When the play ends [it] is like you were not alone feeling all those things [watched a theatre performance].

Sometimes a particular song might come on and you can see the meaning it has to thousands of other people not just yourself so you’re no longer alone [live musical performance].

Other people can feel what I feel when I wrote poems [written as a past time activity].

Of note is that the arts *elicit* shared emotional responses, but also that they *facilitate* sharing of emotions. Second, when people share their own art, they experience a sense of connection between themselves and their audience (sub-sub-theme 2.1.2):

It makes me feel connected as I like to show my creations off and talk about them like my most recent make of an ashtray made out of a can that I got a few compliments on [crafts].

I feel the images I produce can be looked at others and interpreted in whatever they see fit and it gives me joy knowing my work is looked upon by others and they take something away from it [done photography, film, videomaking].

When I play for people I can see it in their faces what they are feeling and it’s the connection through my music that has allowed that to happen [played a musical instrument or sang].

Finally, sub-theme 2.2 highlights how the arts can provide people with a sense of shared purpose:

I am in a place with lots of different people all there for the same reason, to appreciate the performance and share the same moment [watched a theatre performance].

We are all on the same wavelength. A desire to paint better [painting, drawing, printmaking].

As well as the shared purpose of engaging in the activity, this sub-theme also captures wider shared purposes, connected with giving to or supporting others:

I live in a sheltered housing complex and we meet once a week to knit and crochet items which are then sold to raise funds, or given to a charity organisation [crafts].

Working to a common goal, give unity. Supporting and encouraging one another. It helps to bring the best out in people [practised, rehearsed or performed in a play, opera, etc.].

In sum, the second theme captures how arts provides people with shared experiences, including shared emotions, connections between artist and audience, and a sense of shared purpose.

#### Theme 3: facilitating commonality and belonging

The respondents identified that arts engagement, particularly live music and theatre, allows them to feel part of something bigger than themselves (sub-theme 3.1). This is first reflected in feeling part of something, such as an event or atmosphere:

It normally involves large numbers of people and you feel you’re part of one single organism opposed to just alone. It puts you in the zone and you feel a connection with those around you [live musical performance].

Book clubs make me feel incredibly connected with those around me as due to the vibrance [sic] in the actual books makes me potentially feel interconnected with the book and my surroundings [attended a book club].

Watching a film with others we all enjoyed at a drive-in movie surrounded by likeminded people all singing along in their own cars was really enjoyable and made me feel part of the experience [watch a film or drama at a cinema or other venue].

As well as being part of an atmosphere or event, this sub-theme is also connected to the idea of ‘belonging’ as part of a wider community:

I read, watch videos and do searches about it online and through social media, and through this I have seen that there are many people that like me like arts and crafts and also are similar to me in other aspects, which I didn’t know before. Usually I don’t really manage to connect in meaningful ways with others because I feel and am perceived as too different from the people I am around. So even though I have not really personally connected to the arts and crafts people, I feel as part of the community and I have hope that when I feel ready I will be able to join said community in a more active way [crafts].

Rehearsing a play demands that you connect with your fellow performers. We aim to invoke emotion in our audience and so we have to connect with each other in order to feel those emotions ourselves. It’s a deeply collaborative process, one which cannot achieve its full potential without connecting with each other. Even outside of rehearsals, you become a team - a family, even. It’s a wonderful feeling of belonging [practised, rehearsed, or performed in a play, opera etc.].

I play in the village Brass band. You feel like a team and a vital part of the community [played a musical instrument or sang].

Within this sub-theme, the arts were also perceived as a way of being near to other people and times (sub-sub-theme 3.1.1):

Being [in] a room full of people surrounded by art and just appreciating it [been to an exhibition, museum, or collection of art].

Listening to music that I listened to in the [19]60s makes me feel connected to all my friends I went dancing and going to concerts with in my youth [listened to recorded music].

It’s the only time I get a chance to spend time with family and friends so it’s a great activity to do with people [watch a film or drama at a cinema or other venue].

Of note is that this proximity could either be direct (i.e., nearness to others, spending time with others) or indirect (i.e., closeness to other times and to people, including oneself, in the past).

The second and third sub-themes reflect how the arts facilitate feelings of similarity between people. Sub-theme 3.2 captures how the arts, particularly live music, connect people with common interests specific to particular arts activities:

Literature is my greatest love so when I’m at a literary event I feel like I’m in the sort of company that constitutes ‘my tribe’ in the world, the people with whom I have the most in common [been to a book or reading event].

I feel that when I’m at a craft fair other people are as interested as me therefore we have a connection [been to a crafts convention or show].

It makes you feel connect[ed] as there are so many likeminded people there with you who obviously share a love of the band’s music and know as much about them as I do and you feel like you belong to a club [live musical performance].

For some participants, the shared common interests provide a means of connection that might otherwise not feel possible, a finding mirroring sub-sub-theme 1.2.2 (negating pressures of social interaction):

I’m quite an awkward person while meeting new people but as soon as I meet another dancer it’s like I feel instantly connected to them because we have something in common! And when I dance with someone new it’s like magic on the stage because I’m doing what I love with someone who loves it too! Nothing makes me feel more connected to a person then dancing with them does! It’s a truly special feeling [practised, rehearsed, or performed dance].

As well as connecting people with arts-specific common interests, sub-theme 3.3 reflects how the arts bring like-minded people – with common tastes, opinions, and beliefs – together more generally:

I play in the worship team at my local church and the time of worship is a coming together of likeminded people, praising God. So we do feel connected with each other [played a musical instrument or sang].

I think how someone connects with a story/character says a lot about them and I like to engage with people who feel the same way towards a certain story/character as I do [read].

I went to the theatre to see a drag queen. Everyone in the audience was accepting and likeminded which made me feel more connected to others [watched a theatre performance].

In sum, the third theme captures how the arts facilitate feelings of commonality and belonging, including through being part of an event or activity, feeling a sense of belonging, being close to others, and through the sharing of arts-specific shared interests and connections with likeminded people.

#### Theme 4: facilitating collective understanding

The fourth theme focuses on the ways in which the arts facilitate learning from and about other people in ways that foster social connections. Sub-theme 4.1 reflects how the arts, particularly crafts, allow people to learn new skills and knowledge with and from others:

My sister and I went to a pottery class last month, and as well as learning new techniques together, we managed to catch up, give each other advice, and take a keepsake of our special day [crafts].

I work alongside others and can throw ideas and solutions at each other. They make me better and I make them better. We complement each other [done photography, film, videomaking].

The connection when someone enjoys the same books that you do and when your child takes an interest in some of them and asks that you read them at bedtime, then starts asking questions about the content because they’re taking it all in and want to understand it all better. It’s all heartwarming, and the world suddenly feels a little less lonely [read].

Linked to this, sub-theme 4.2 illustrates how the arts allow people to relate to others:

I suppose it’s usually when the speaker or author is someone I admire or respect. Often their experiences and what they write about reflect my own experiences and I feel less alone [been to a book or reading event].

Relating to their stories. Makes me feel like I’m not alone. Sharing stories. Help feel normal [listened to audiobook or podcast].

It stops you feeling so alone, when someone else can describe a situation the same way you experience it [read].

Of particular note here is that the arts do not need to be ‘in person’ to offer opportunities for relating to others. In fact, the three examples shown here illuminate how the arts facilitate connections at an emotional and experiential level, allowing for relatedness through the artistic product.

Additionally, the arts, particularly reading, facilitate a means of learning *about* other people, places, and times (sub-theme 4.3):

Being outside and exploring with someone particularly if it has a historical interest where you can feel connected to the people before us [been to an exhibition, museum, or collection of art].

I feel I connect with other people through photography. I get to spend time with people. Listen to their stories. I try to capture their emotions, what they’ve been through and what they are currently going through [done photography, film, videomaking].

The podcasters are talking about something that interests both them and me. Listening to the same podcasters regularly makes me feel like I am getting to know them [listened to audiobook or podcast].

It opens up new worlds and new ideas. It helps me to understand the lives and experiences of other people [read].

Here, we see the arts connecting people directly (e.g., through photography) but also indirectly, such as through podcasts or books, or through connecting to people from other times through an artistic activity.

The final two sub-themes within this theme illustrate how the arts foster connections through *differences*. Sub-theme 4.4 captures this in respondents’ descriptions of how the arts expose people to different cultures, bring together people from different backgrounds:

They usually involve people from various backgrounds, of different ages but all with a love of music in common so instil a sense of common ground, no matter the otherwise diversity of the attendees [been to a live musical performance].

You get such a variety of people at art venues of museums, all sharing an experience which makes me feel like part of the wider world [been to an exhibition, museum, or collection of art].

A shared love of music, sometimes a specific type, shows that regardless of age or other interests, there is a common bond that can be explored … [It] may make someone realize that people who may seem very different, may not be so different after all [listened to recorded music].

Here, we see social connectedness linked with being ‘part of the wider world’ through exposure to diversity. Linked with this, sub-theme 4.5 captures how the opportunity to be exposed to different viewpoints and opinions was also expressed as connecting:

It gives you a chance to see other perspectives and hear new ideas, seeking those things out is one of the most human things you can do [attended a book club].

This makes me think about how other people interpret and see things. It makes me see how we can see things differently. Like an abstract sculpture can be seen to appear very different to me than the person who created it, however I feel connected as we can both see something united [been to a crafts convention or show].

I find drawing and painting a relaxing and rewarding experience whether alone or as member of a group. It is amazing that a number of people can draw or paint a specific scene or object but their interpretation will appear different in almost every case and can become the topic of in depth conversation [done painting, drawing, printmaking].

In sum, the fourth sub-theme captures how the arts facilitate learning from and about other people, building social connectedness through learning from and with others, relating to others, learning about others, connecting with different people and cultures, and connecting with different viewpoints.

## Discussion

This article has addressed three research questions pertinent to the role of the arts in facilitating social connectedness for public social health. The first question related to the extent to which adults in the UK perceive a link between their arts engagement and feelings of social connectedness. Our findings show that over 80% of the respondents reported that their engagement in the arts makes them feel socially connected at least ‘a little’. This finding is expected given the previously cited evidence that the arts can support social connections, although the evidence base to date has been most extensive in research with older adults [17–20]. This study extends the evidence base through a wider sample, based on key demographics of the adult UK population and representing a much wider range of adult age groups.

The second question focused on which forms of arts engagement are most reported as linked with feelings of social connectedness. While previous research has tended to focus on certain arts activities – notably museum/gallery attendance [21–23] and music [25, 28–30] – the current study demonstrates a wider range of activities that respondents report connects them socially to others. Both live musical performance and visiting an exhibition, museum, or gallery were among the top five listed activities, but these sit alongside activities less reported in the existing literature including live theatre performance, watching a film or drama at the cinema, and reading. As well as expanding the range of arts activities that can be seen as socially connecting, these findings emphasise that it is not only participatory activities – those where a participant makes or creates art themselves – that achieve this connection. This is an important finding, given that existing evidence based on intervention studies often focuses predominantly on participatory activities, such as choral singing [31], dance [38], and textile crafting [40]. Here, the activities reported as most connecting were more ‘receptive’, undertaken – with the exception of reading – at arts venues outside of the home. The implications of this in light of the COVID-19 pandemic are important, as activities such as concerts, theatre productions, and cinema screenings have been closed or reduced. Further research will be required to identify whether and how the arts support social connections during and after the COVID-19 pandemic.

The third research question focused on *how* arts engagement is perceived to facilitate feelings of social connectedness. To answer this, we conducted a large-scale qualitative analysis of data that resulted in four overarching themes: the arts support social connections through facilitating social opportunities, facilitating sharing, facilitating commonality and belonging, and facilitating collective understanding. The first theme – social opportunities – speaks to the ways in which the arts can support or enhance social networks. This includes explicitly extending social contacts through meeting new people and making new friends, but also opportunities simply to interact with other people, either in general or through specifically discussing an arts activity. In this theme we see overlaps with what Wang and colleagues refer to as ‘network quantity’ in their work on social isolation in mental health, or in other words ‘the number of people in someone’s social network, [the] number or frequency of someone’s social contacts over a period of time’ [62]. Of particular note here are the sub-sub-themes conversing about art (1.2.1) and negating pressures of social interaction (1.2.2). Both imply the role that the arts can play in providing a catalyst for social interaction (e.g., having a conversation) but also a means of connecting with others that can be experienced as more accessible or less pressured than ‘typical’ social interaction (e.g., being able to have a ‘silent conversation’). Indeed, the non-verbal nature of some forms of arts engagement has been highlighted in other research as important for connecting people [27].

The second theme – facilitates sharing – encapsulates the ways in which respondents reported that arts engagement leads to shared experiences. This links with Hare-Duke and colleagues’ conceptual framework for social connectedness in mental disorders, and in particular their dimension of ‘closeness’ [15], or feeling close to other people. This closeness appears to be facilitated by the shared experienced of an arts activity itself (e.g., sharing a cinema screening) but also by feelings of shared purpose and emotions. Given that loneliness is emotional as well as social [2], it may be that arts engagement supports feelings of social connection through facilitating emotional closeness and feelings of emotional intimacy with other people.

In the third theme, the arts emerged as a means of eliciting feelings of commonality and belonging, a finding echoed in O’Rourke and Sidani’s operationalization of social connectedness [13]. Indeed, that the arts can support a sense of belonging is highlighted by other research [27, 33, 63–68]. This theme also highlighted the importance of being near to other people, either directly (e.g., in person) or indirectly (e.g., feeling connected to old friends when listening to certain pieces of music), making the important point that connection through the arts does not require people to be *physically* together. For some respondents, the notion that others had or could engage with the same art form could also instil feelings of social connections. This finding is mirrored elsewhere, with museum attendees voicing that they felt connected to others who may have viewed the same museum exhibits [69]. Similarly, it has been previously suggest that arts engagement can reduce feelings of loneliness through acting as a social surrogate [32, 33, 70]. Therefore, not only can the arts provide social connections despite an absence of physically proximity to others, but they may also enable people to create the concept of another person to whom they feel connected. Finally, this theme related to ‘being at one’ through, for example, singing together as part of the audience at a live music performance. We can see a link here with previous evidence that singing, for example, can promote fast cohesion among people who are not familiar with one another [29], a point reinforced by the ways in which respondents in this study reported feeling ‘at one’ with others in large audiences.

This third theme also focused on how arts engagement can elicit feelings of commonality between people, evident through the arts connecting people with common interests, tastes, and opinions. The dimension of ‘identity and common bond’ identified by Hare-Duke and colleagues is relevant here: ‘believing that one shares important characteristics with other people or members of a group’ [15], as well as their notion of ‘social acceptance’, or the ‘perception of being an accepted member of a particular group or community’ [15]. Indeed, theories of social identity posit that people’s personal identification with social groups are important for health [71], more so than social contact [72]. The arts have been shown to create this sense of social identity, or group identification, in this study and elsewhere [73, 74]. Of note is that social identity in this study emerges as both directly related to an arts activity (sub-theme 3.2) but also as wider than the arts activity itself (sub-theme 3.3), again demonstrating how the arts can support social connections both directly and indirectly.

Finally, the fourth theme – facilitating collective understanding – reflects how the arts supports social connections through allowing people to learn from and about other people. This includes ‘valued relationships’ [15] including learning new skills with others, and learning about others. This finding echoes the ideas of Maidment and Macfarlane (2009), who discuss how reciprocity of learning with others in a crafts club enhanced social connectedness [75], linking this with Siegrist et al.’s concept of ‘social productivity’, where people spend effort supporting others in socially valued activities [76]. Of particular note here are the two sub-themes that reflect feelings of connection through *difference*, either by connecting with different people and cultures, or through connecting with people who hold different viewpoints. These are perhaps the most unique among the sub-themes, illuminating how the arts bring together people who may be different to one another, but who share an interest in the same activity. This can lead to connection through, for example, giving a sense of being part of the ‘wider world’, through a realisation that people whom we think of as different may in fact not be so different, and through exposure to different opinions and ideas on the same arts activity. Indeed, researchers have highlighted that, while museums have historically promoted specific ideologies, they can also act as safe spaces where members of communities can share a diversity of opinions and be exposed to other cultures [77–79]. Furthermore, exposure to the arts has also been shown to increase levels of tolerance to other viewpoints, with research finding that, through discussing works of art, medical students could develop an ability to accept multiple interpretations and increase their levels of empathy [80]. This article extends these findings beyond viewing artworks and visiting museums, with arts activities including attending book clubs, engaging in photography, and listening to audiobooks enabling users to feel a connection to others with differences of opinions. As a result, the arts may act as ‘deliberative spaces’, ideal environments where individuals ‘can discuss and debate common concerns, access a wide range of information, and reflect and revise their understanding of issues’ [81].

**Fig. 1:**
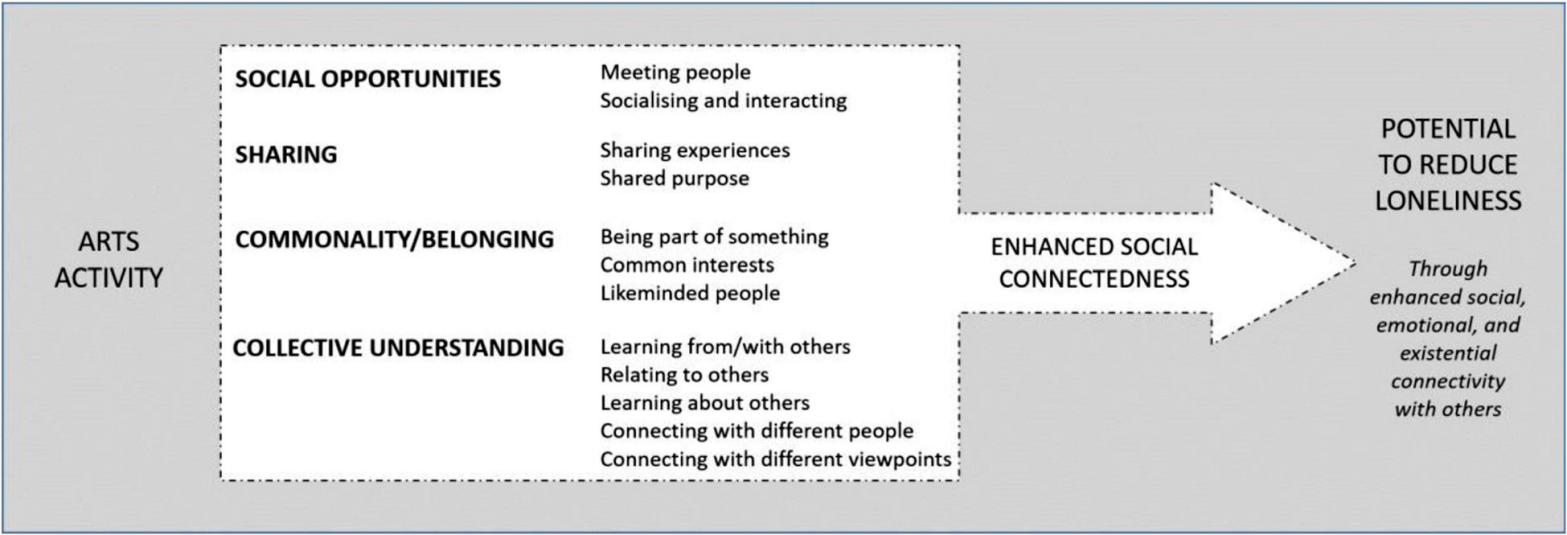
How arts engagement is perceived to facilitate feelings of social connectedness

Taken together, the four themes and their sub-themes present a complex account of how arts engagement can support social connectedness, as presented in Fig. 1. Two points are of note. First, the ways in which the arts support social connections should be understood as complex. This complexity is evident not only in the breadth of sub-themes, which illustrate wide ranging means of social connection through the arts, but also in the overlap between themes and between sub-themes. Many of the survey responses were coded to multiple codes in the first step of the analysis, meaning that respondents were identifying multiple points of connection in their responses. It should not therefore by inferred that an arts activity facilitates one form of connectivity only, but rather that multiple forms of connectivity can occur simultaneously. Second, respondents were asked to write about the activity they felt *most* connected them to other people. This means that the evidence presented in this study focuses on the ‘optimal’ connective properties of the arts, and that it is specific to a particular activity as selected by individuals. It is not the case that all arts activities will facilitate all four modes of connection, and it should be assumed that the choice of arts activity and the ways in which it supports connection (or not) are both highly idiosyncratic within and between individuals. In music, for example, a recent meta-ethnography [82] made explicit the importance of taking into account individual needs within specific individual circumstances when understanding how participatory music engagement supports mental wellbeing, and the same is likely to be the case with understanding how the arts support social connectedness.

## Conclusion

Given that loneliness is linked to mortality as well as poorer physical and mental health outcomes [4–6], finding ways to prevent or reduce it is important for social public health. Social connectedness has been proposed as a means of reducing loneliness [15], focusing attention on what people can do to build meaningful social connections [14]. This article has demonstrated that, in a large sample matched to key demographic characteristics of the UK population, over 80% of respondents reported that arts engagement makes them feel connected to others, particularly live music performance, theatre performance, watching a film or drama at the cinema or other venue, reading, or attending exhibitions, museums, or galleries. This evidence suggests that people at risk of or experiencing loneliness can be directed towards arts activities as a potential means of developing social connections. It also reinforces the evidence base on the role of the arts in health and wellbeing [83], reminding us of the potential health implications of the current COVID-19 pandemic which has shut arts venues, prevented professional and amateur arts activities, and forced a skills exodus from the arts profession. Further research will be required in order to understand how people have used the arts to connect with others since the start of the COVID-19 pandemic, and how this has changed as the situation has developed. Finally, in exploring *how* the arts can support social connections, the findings have presented a rich tapestry of pathways. These speak to social connections that have the potential to mitigate emotional, social, and existential loneliness [2, 3], and provide evidence for those looking to commission arts activities that enhance social connectedness as well as those who design and deliver them.

This research is limited, first, by an inherent bias in the sample, in that all respondents providing a response to the three included survey questions had, previously in the survey, already identified that they engaged in arts activities at least once in the past 12 months. Further, levels of loneliness and depression were higher than expected in the sample, potentially influencing the ways in which respondents reflected on their use of the arts for social connectedness. Second, the analysis process was by necessity interpretative. To add validity the themes were cross-checked by two authors at three time points during the analysis process (10, 50, and 100%) and the final overarching themes and sub-themes were agreed by three authors. Working with such a large qualitative dataset was resource heavy, but saturation was taken to only be achieved once all possible perspectives were included, a point reinforced by some of the smaller yet still pertinent sub-sub-themes. Third, the data were limited by the mode of response in the survey, which was ‘one-time’ and anonymous. While this did not allow for any clarification or probing of respondents’ answers, nor for further exploration with those who reported that their arts engagement was *not* associated with feelings of social connectedness, it did allow for large-scale qualitative data collection in an efficient manner. The collection and analysis of ‘big qual’ [84] data in the field of arts and health is important as a means of understanding the complexity of how large groups of people engage with the arts and how this interacts with their perceived health. In adopting this approach here, this article provides robust evidence of how the arts support social connectedness among a large sample of adults in the UK.

## Supplementary Information

**Additional file 1.**

**Additional file 2.**

## Data Availability

The datasets used and/or analysed during the current study are available from the corresponding author on reasonable request.
