# Supplementary material for: Arts engagement supports social connectedness in adulthood: findings from the HEartS Survey
Source: BMC Public Health. 2021 Jun 24;21:1208. doi: 10.1186/s12889-021-11233-6 (PMC8221987; doi:10.1186/s12889-021-11233-6)
Supplement: Supplementary file 1 — Additional file 1. [file 12889_2021_11233_MOESM1_ESM.pdf]

Perkins R, Mason-Bertrand A, Tymoszuk U, Spiro N, Gee K, and Williamon A (2021), Arts engagement supports social connectedness in adulthood: findings from the HEartS Survey, *BMC Public Health*.
doi: 10.1186/s12889-021-11233-6

SUPPLEMENTARY TABLE 1 | Sociodemographic, economic, and health and wellbeing characteristics of the respondents.

***Sociodemographic and economic characteristics* (N=5892)**		
**Age**	*mean*	*SD*
	45.86	16.90
	n	*%*
18-25	775	13
26-35	1221	21
36-45	1021	17
46-55	909	15
56-65	1026	17
66-75	796	14
76-94	144	2
**Gender**		
Women	3027	51
Men	2835	48
Would rather not say	12	0
Other	18	0
**Region**		
Northern Scotland	136	2
Southern Scotland	265	4
North East	216	4
North West	667	11
Yorkshire and the Humber	500	8
East Midlands	440	7
West Midlands	531	9
East of England	559	9
South East	829	14
South West	517	9
London	770	13
North Wales	79	1
South Wales	219	4
Northern Ireland	164	3
**Ethnicity**		
White British or Irish	5032	85
Any other White background	264	4
Mixed ethnic backgrounds*	136	2
Asian ethnic backgrounds*	275	5
Black ethnic backgrounds*	146	2
Any other ethnic background	39	1
**Education**		
No formal qualification	213	4
Other vocational and foreign qualifications	691	12
GCSE, O Level, AS Level – NVQ Level 1-2	1507	26
A level, baccalaureate – NVQ Level 3	1340	23
University degree – NVQ Level 4-5	2137	36
Would rather not say	4	0

**Living status**		
Lone living	1199	20
With partner only	2047	35
With children (with or without partner)	1725	29
With family, house share, and other	893	15
Would rather not say	28	0
**Household income**		
Up to £5,199	233	4
£5,200 and up to £10,399	406	7
£10,400 and up to £15,599	568	10
£15,600 and up to £20,799	604	10
£20,800 and up to £25,999	635	11
£26,000 and up to £31,199	614	10
£31,200 and up to £36,399	405	7
£36,400 and up to £41,599	441	7
£41,600 and up to £46,799	298	5
£46,800 and up to £51,999	311	5
£52,000 and up to £75,999	547	9
£76,000 and above	344	6
Would rather not say	486	8
Social connectedness, loneliness, and depression		
**Social connectedness**	*mean*	*SD*
Social connectedness scale, score 0-75	41.62	15.37
**Loneliness**		
Score (UCLA, 3-9)	5.27	1.96
	n	*%*
Cases (UCLA, cutoff ≥ 6)	2722	46
**Depression**	*mean*	*SD*
Score (CES-D 8, 0-8)	4.49	2.04
	n	*%*
Cases (CES-D 8, cutoff ≥ 3)	4600	78

* Ethnicity: any mixed background includes White and Black Caribbean, White and Black African, White and Asian, and any other mixed background; any Asian background includes Indian, Pakistani, Bangladeshi, Chinese, or any other Asian background; any Black background includes Caribbean, African, and any other Black background.
