# Supplementary material for: Arts engagement supports social connectedness in adulthood: findings from the HEartS Survey
Source: BMC Public Health. 2021 Jun 24;21:1208. doi: 10.1186/s12889-021-11233-6 (PMC8221987; doi:10.1186/s12889-021-11233-6)
Supplement: Supplementary file 2 — Additional file 2. [file 12889_2021_11233_MOESM2_ESM.pdf]

Perkins R, Mason-Bertrand A, Tymoszuk U, Spiro N, Gee K, and Williamon A (2021), Arts engagement supports social connectedness in adulthood: findings from the HEartS Survey, *BMC Public Health*. doi: 10.1186/s12889-021-11233-6

SUPPLEMENTARY TABLE 2 | Frequency and indicative quotes for each (sub-)sub-theme, presented according to arts activity for four overarching themes. Quotes are verbatim.

Overarching theme 1 Facilitating social opportunities

Sub-theme/sub-sub-theme	*Arts activity*	*Frequency*	*Indicative evidence*
1.1 Meeting new people	Been to a live musical performance	46	Once I went to a music festival and I ended up with many newfound friends as we had similar music tastes
	Watched a theatre performance live	7	Two of my sisters are professional singers and actresses so I spend a lot of time in this environment and always meet new people through it
	Watched a film/drama at cinema/other venue	1	Meet new people
	Read as a past-time activity	2	I meet other people and hear their views
	Been to an exhibition, museum, etc.	14	It’s lovely to meet new people and mutually share perspectives
	Played a musical instrument or sang	6	I am part of a small choir and I have made new friends through this activity. We all get on well together as we share our love of Christian music
	Deliberately listened to recorded music	1	I have met likeminded people through this in the past
	Listened to audio books or podcasts	1	Meeting new people, engage other things to improve the community
	Attended a book club	11	You meet new people and discuss likes and dislikes of certain books
	Been to an event connected with books/reading	8	I can meet people who enjoy the same things as me and make new friends
	Done any form of crafts	7	It gets me outside the home to meet new people and make new friends
	Been to street art, public art displays, etc.	5	[Because] anyone can come there you meet a lot of nice and interesting people
	Done photography, film, video making, etc.	1	Because it is something I am passionate about, something I enjoy doing and make a living out of. I’ve been interested in photography since I was about five, when I played around with my dad’s camera and took pictures of random things, myself and my parents. Ever since then I’ve been

			getting better cameras, getting more skilled at my interest and job, and have made lots of life-long friends due to it
	Written as a past-time activity	2	I've met a lot of people through it
	Practised, rehearsed, or performed dance	5	It is sociable and a good way of meeting people
	Done painting, drawing, printmaking, etc.	4	Because it's actually how I met most of my friends. I was in the school library drawing when a group of four people with sketchbooks came up to me and started complimenting my work. We kept talking and now we're all best friends
	Written or created music	0	N/A
	Practised, rehearsed, or performed a play, etc.	2	I participate in plays and musicals within my local parish. This is great way to meet new people with others with similar interests
	Watched a dance performance live	2	Because it lets you meet new people
	Been to a craft convention, show, or fair	5	Meeting new and interesting people and hearing about their interests
	'Other'	0	N/A
1.2 Socialising and interacting with others	Been to a live musical performance	127	People smile and interact with others at the venue. A good sense of community
	Watched a theatre performance live	37	Sitting in an audience and interacting with other people. Big groups of people getting together to enjoy the same thing.
	Watched a film/drama at cinema/other venue	34	It makes me feel socially connected so I can have conversations with people I know, it makes me feel included and enables me to engage with others. I enjoy the cinema and enjoy getting away from my daily routine.
	Read as a past-time activity	8	You can enjoy a book together
	Been to an exhibition, museum, etc.	25	As a public place, one cannot help being surrounded by and interact with other people
	Played a musical instrument or sang	15	Belonging to several choirs gives me the opportunity to engage with the people I am performing with and to the people I am performing to
	Deliberately listened to recorded music	4	[Because] we interact more listening to music
	Listened to audio books or podcasts	8	I feel connected to the hosts of the podcasts. Sometimes I will even talk back to some comments!

	Attended a book club	30	The interaction of the others
	Been to an event connected with books/reading	25	It brings everyone together as a social event. It's a good opportunity to chat and build relationships with others
	Done any form of crafts	11	It's a knitting group with a wide age range. The founder set it up as a group to help with mental health issues. It's sociable, inspirational and makes what could be a solitary pastime into one to be shared
	Been to street art, public art displays, etc.	14	Visiting archaeological sites provokes conversation and interaction with others
	Done photography, film, video making, etc.	13	The interaction between us when using the camera has provided many enjoyable moments recorded as memories in the photos
	Written as a past-time activity	1	I write to my pen friends so that makes me feel connected to them
	Practised, rehearsed, or performed dance	25	I dance Ballroom, Latin and Le Roc jive with my wife and others as often as we can. Despite having some mobility issues we enjoy the social aspects of dancing with other partners. We are both in our mid 70's and will continue to enjoy dancing as long as we are able to participate
	Done painting, drawing, printmaking, etc.	6	Paint with my grandson and daughter. It makes us laugh and I feel less alone
	Written or created music	3	Is very sociable
	Practised, rehearsed, or performed a play, etc.	9	We are interacting all the time so must be aware of what each person is doing in relation to oneself. This brings a spiritual non spoken bond as you are willing the others to get everything right and on time
	Watched a dance performance live	5	When watching street shows you are in a crowd of strangers and often find myself talking to the person next to me, this often happens at the Edinburgh Festival which has lots of street shows
	Been to a craft convention, show, or fair	19	There are a lot of people there and it is great for socialising
	'Other'	4	Spiritually and socially connected with those I meet with whilst engaging in Christian worship
1.2.1 Conversing about art	Been to a live musical performance	26	Because I normally go with other people who share my love of music, or of the artist we are seeing, and we get together before and after the event to discuss the performance and other artists/genres of music

Watched a theatre performance live	54	I get to spend quality time with family/friends and get to talk about it afterwards which gives me a good connection with
Watched a film/drama at cinema/other venue	104	Feel everyone is open to express the opinions on how the film was and it good then to help you to see what other people's interests are
Read as a past-time activity	64	It starts conversation and discussions and can also start debates
Been to an exhibition, museum, etc.	69	Admiring things with people and going to museums sparks conversation which helps me to connect
Played a musical instrument or sang	10	It gives you a fascinating glimpse into other's personalities. E.g. someone being rebellious by throwing chords that shouldn't, and doesn't, work into a jamming session. As the sessions go on you tend to relax and talk freely
Deliberately listened to recorded music	23	We talk about the music we are listening to and talk about stories when we first heard the song and stuff
Listened to audio books or podcasts	18	This activity makes me feel connected to other people because they will understand the same perspective as me. Listening to an audio, everyone has their own opinion which becomes a discussion
Attended a book club	63	It gives me the opportunity to converse with other people, to exchange ideas and review conceptions
Been to an event connected with books/reading	11	Discussing how different people interpret the same piece of literature is a great way to learn a lot about someone and connect with them. To understand how their emotions were affected by words
Done any form of crafts	27	It's a good topic that interests me to chat about
Been to street art, public art displays, etc.	28	Street entertainment seems to create camaraderie between strangers, especially when it is unexpected and catches people by surprise. The public will often discuss what is going on with those around them, even if they have never met each other before
Done photography, film, video making, etc.	19	It allows you to collaborate with other artists, merging your ideas together. In addition, you are able to give each other feedback which will help develop images
Written as a past-time activity	10	People share their appreciation or critique with me

	Practised, rehearsed, or performed dance	1	We all have the ability to give new suggestions, helping each other out where needs be. It’s a good experience where we can all feed off each other’s good ideas and improve together
	Done painting, drawing, printmaking, etc.	11	Because it's actually how I met most of my friends. I was in the school library drawing when a group of four people with sketchbooks came up to me and started complimenting my work. We kept talking and now we're all best friends
	Written or created music	3	Express the ideas that I have inside my head transform them into music, share with other people and get their feedback is a great way to connect with people
	Practised, rehearsed, or performed a play, etc.	0	N/A
	Watched a dance performance live	3	I can review the act with others and socialise better
	Been to a craft convention, show, or fair	10	Have a look at all the things people have made and chat to them about them
	'Other'	4	Its subject was of extreme interest to others which encourages conversation with people you would not have conversations
1.2.2 Negating pressures of social interaction	Been to a live musical performance	4	Because you can be together without having to engage with anyone and still share in the music and the feeling
	Watched a theatre performance live	1	[I] get to enjoy the company of friends without having to worry about chatting and doing anything the whole time as you have something to watch
	Watched a film/drama at cinema/other venue	2	The immersive quality and independence from a group whilst with others
	Read as a past-time activity	1	Because reading allows you to put yourself into a story which you can share with others without feeling to much social pressure
	Been to an exhibition, museum, etc.	1	I am a mother of a large family. However, I consider myself a loner. Going to museums and such, when I am alone, makes me feel connected to the people who created the pieces. It's akin to having a silent conversation with the artist. I enjoy this
	Played a musical instrument or sang	0	N/A
	Deliberately listened to recorded music	1	Listening to music is a good way to break the ice if you don’t know them

Listened to audio books or podcasts	1	Because I feel more confident being with others
Attended a book club	0	N/A
Been to an event connected with books/reading	1	You are with people who are interested in the same thing as you which makes it easier to interact with people you don't know
Done any form of crafts	0	N/A
Been to street art, public art displays, etc.	1	We usually do it as a family day out. Unlike gigs, where I tend to feel disconnected from the rest of the audience as I am typically outside my comfort zone due to noise, crowds, socialising with acquaintances/strangers etc.
Done photography, film, video making, etc.	0	N/A
Written as a past-time activity	1	I have not really felt close to people as I have anxiety and this helped
Practised, rehearsed, or performed dance	0	N/A
Done painting, drawing, printmaking, etc.	1	Drawing classes are a good way to socialise in a low stakes way, and sharing work online is an accessible way to get feedback and participate in the artistic community
Written or created music	0	N/A
Practised, rehearsed, or performed a play, etc.	0	N/A
Watched a dance performance live	0	N/A
Been to a craft convention, show, or fair	0	N/A
'Other'	0	N/A

Overarching theme 2 Facilitating sharing

Sub-theme/sub-sub-theme	*Arts activity*	*Frequency*	*Indicative evidence*
2.1 Sharing experiences	Been to a live musical performance	238	The performance makes me feel more connected to people, like we're sharing something
	Watched a theatre performance live	110	It gives you a sense of common ground with those you are experience the theatre with and the show helps you engage and connect with those around you
	Watched a film/drama at cinema/other venue	87	Cinema is a shared experience despite being in the dark and no having contact with anybody
	Read as a past-time activity	21	It stops you feeling so alone, when someone else can describe a situation the same way you experience it
	Been to an exhibition, museum, etc.	39	Being able to share thoughts and opinions about what we are seeing and continuing to discuss them afterwards, also looking at the photos we have taken while there. Last week we visited a museum in Croatia which spanned 4 floors and was devoted to just one exhibit on the top floor. This involved being taught about the statue, having our bodies cooled to the correct temperature to view it and chatting about what we were experiencing. Later we looked at the literature and snaps and kept talking about the whole experience and the different ways that we viewed it. It was a great group experience
	Played a musical instrument or sang	55	I sing in a choir run by the Tenovus Cancer Care charity which is a Welsh charity for anyone affected by cancer - I had sarcoma. There are ninety of us, and this is just 1 out of 18 such choirs in Wales. We are very good and get lots of gigs. We watch out for one another and raise funds for Tenovus. It's upbeat but also quite emotional at times. I couldn't live without it

Deliberately listened to recorded music	51	We feel more connected because we get to dance with each other and encourage each other to dance. It makes for great memories. At prom my group of friends were the only ones on the dance floor and we spent the majority of our time there. We had so much fun
Listened to audio books or podcasts	11	The feeling that others are listening to the same content as me
Attended a book club	16	Because you discuss a shared experience about reading a book and this gives insight into other people's opinions, history and likes and dislikes
Been to an event connected with books/reading	6	Sharing the experience, listening to others opinions
Done any form of crafts	41	Others doing the same activity and comparing and conversing with humour
Been to street art, public art displays, etc.	15	It is the shared sense of witnessing something. It gives it a certain sense of permanence, and timelessness. Going to see something as evocative and inspiring as the reclining Buddha in Bangkok makes you think of all the other people that have stood where you are. It makes you contemplate how they felt. It gives you a far greater depth of perception and to my mind increases your human consciousness
Done photography, film, video making, etc.	20	It's something I do to spend quality time with my niece and get out to a place where other people are. Seeing people living their lives and capturing nature at its best is part of what makes me feel connected
Written as a past-time activity	4	I like to share my experiences
Practised, rehearsed, or performed dance	14	It is a collaborative experience where we all work together to create a dance routine. We also have fun whilst dancing
Done painting, drawing, printmaking, etc.	16	Currently I do all these activities with my children so it is an activity for us to do together
Written or created music	9	I'm usually doing this in a party setting with my friends so I'm already connected and the act of creating something together amplifies that
Practised, rehearsed, or performed a play, etc.	14	because I’m in the play with other people , we rehearse together and get to know each other then perform in front of a group of people
Watched a dance performance live	9	Because everyone is watching the same performance, mesmerised and happy. Whereas, music for me is more solitary whilst dance is more social
Been to a craft convention, show, or fair	3	Being able to share experiences and learn new skills

	'Other'	10	Because the people that go there have mental health difficulties like me
2.1.1 Sharing emotions	Been to a live musical performance	203	Sometimes a particular song might come on and you can see the meaning it has to thousands of other people not just yourself so you're no longer alone
	Watched a theatre performance live	83	When the play ends [it] is like you were not alone feeling all those things
	Watched a film/drama at cinema/other venue	31	Many other people that also watch the films enjoy them as much as I do
	Read as a past-time activity	6	I sometimes feel disconnected from life around me. Through reading I feel more connected emotionally and find it relaxing, enjoyable, thought provoking and good for mental wellbeing
	Been to an exhibition, museum, etc.	14	It's all about reacting to what you are seeing and it causing an emotional response and this is better shared with others
	Played a musical instrument or sang	24	Brings about a sense of connected joy
	Deliberately listened to recorded music	17	Music connects people. It has no language borders. You listen to a song or a music piece and you know it has been heard by many different people from different backgrounds. When you go see your favourite artists live and you hear everyone singing back to them and you know, whatever you felt listening to that song, millions of others felt as well
	Listened to audio books or podcasts	5	Helps me connect with the feelings of others and escape the world
	Attended a book club	6	Share together feelings of the book and experiences in life and it's welcoming and friendly
	Been to an event connected with books/reading	5	I see other people enjoying themselves
	Done any form of crafts	4	The expression of peoples emotion and thought through their work connects me without having to speak
	Been to street art, public art displays, etc.	11	Connected because of the feeling of enjoyment that is also felt by others
	Done photography, film, video making, etc.	1	My goal in every wedding is to tell not just a love story but a story About Love, and that's the difference. At the end it's like reading a good book with a happy ending and not only pictures. A story can be told in words placed in sentences by a storyteller, me as a photographer I see words depicted in every wedding. So here are some words for my photography just to explain my style!!! Journey, faith, life, travel, partner, special, dream, frame, trust, destination, loving, story, artistic, party, dance,

			friend, weird, different, wander, capture, unique, documentary, shooting, emotion, imagine, bright, enjoy, crazy, professional, fine art, happiness, believe, observer, pretty, hugs, honest, adventure, heart, natural, true, fun, modern, romance, forever
	Written as a past-time activity	11	Because other people can feel what I feel when I wrote poems
	Practised, rehearsed, or performed dance	9	We connect emotionally as we have a lot in common and everyone is passionate about what they are doing
	Done painting, drawing, printmaking, etc.	7	I attend a regular art group for which I have friends. It also provides me with an outlet to talk about my problems and get out of frustration through art
	Written or created music	14	Because I understand the emotions and feelings that go into song writing
	Practised, rehearsed, or performed a play, etc.	8	I feel connected to the audience, as I am portraying a character they believe in, and come to feel and show real emotions for. When acting, both with fellow actors and the audience, we all feel free to express feelings and emotions we may not in day to day life. It breaks down the barriers of social norms, in that moment, it's the moment that matters and nothing else
	Watched a dance performance live	8	Because it is an art form about beauty and expression and everyone can feel that when they watch it
	Been to a craft convention, show, or fair	0	N/A
	'Other'	4	I feed off of the energy the audience give off, and they feed off of the energy and excitement the bands and I give
2.1.2 Sharing art with others	Been to a live musical performance	0	N/A
	Watched a theatre performance live	1	Sharing art
	Watched a film/drama at cinema/other venue	0	N/A
	Read as a past-time activity	0	N/A
	Been to an exhibition, museum, etc.	0	N/A
	Played a musical instrument or sang	23	When I play for people I can see it in their faces what they are feeling and it's the connection through my music that has allowed that to happen

	Deliberately listened to recorded music	4	Music makes my heart sing feeds my soul makes me so happy I need to share with others
	Listened to audio books or podcasts	2	Because I have a story to tell and people listen
	Attended a book club	0	N/A
	Been to an event connected with books/reading	0	N/A
	Done any form of crafts	23	It makes me feel connected as I like to show my creations off and talk about them like my most recent make of an ashtray made out of a can that I got a few compliments on
	Been to street art, public art displays, etc.	0	N/A
	Done photography, film, video making, etc.	26	I feel the images I produce can be looked at others and interpreted in whatever they see fit and it gives me joy knowing my work is looked upon by others and they take something away from it
	Written as a past-time activity	17	Because I write for other people
	Practised, rehearsed, or performed dance	3	It is nice to show my work for others to enjoy
	Done painting, drawing, printmaking, etc.	12	I take in recommendation of what drawings people want to see
	Written or created music	4	Because they can hear my music and feel what I felt it creates a connection
	Practised, rehearsed, or performed a play, etc.	2	It's where I feel most alive and that shows in my performances and how they affect the audience
	Watched a dance performance live	0	N/A
	Been to a craft convention, show, or fair	0	N/A
	'Other'	4	People read my work and get a lot of enjoyment out of it. Even though I'm not that popular, making anyone smile makes me happy
2.2 Shared purpose	Been to a live musical performance	95	Everyone is there for the same reason, it is like you belong together
	Watched a theatre performance live	11	Because I am in a place with lots of different people all there for the same reason, to appreciate the performance and share the same moment
	Watched a film/drama at cinema/other venue	3	All there for the same reason
	Read as a past-time activity	0	N/A

Been to an exhibition, museum, etc.	4	At an art exhibition everybody is there to admire the artists work. Therefore you have a common objective
Played a musical instrument or sang	3	Because everyone plays their own instruments with different sounds but they all come together to play 1 song
Deliberately listened to recorded music	0	N/A
Listened to audio books or podcasts	0	N/A
Attended a book club	0	N/A
Been to an event connected with books/reading	2	All working together as a creative element
Done any form of crafts	3	I live in a sheltered housing complex and we meet once a week to knit and crochet items which are then sold to raise funds, or given to a charity organization
Been to street art, public art displays, etc.	0	N/A
Done photography, film, video making, etc.	0	N/A
Written as a past-time activity	0	N/A
Practised, rehearsed, or performed dance	0	N/A
Done painting, drawing, printmaking, etc.	1	We are all on the same wavelength. A desire to paint better
Written or created music	0	N/A
Practised, rehearsed, or performed a play, etc.	3	Working to a common goal, give unity. Supporting and encouraging one another. It helps to bring the best out in people
Watched a dance performance live	0	N/A
Been to a craft convention, show, or fair	0	N/A
‘Other’	0	N/A

Overarching theme 3 Facilitating commonality and belonging

Sub-theme/sub-sub-theme	*Arts activity*	*Frequency*	*Indicative evidence*
3.1 Being part of something	Been to a live musical performance	261	It normally involves large numbers of people and you feel you're part of one single organism opposed to just alone.
	Watched a theatre performance live	107	The theatre experience is a positive one with a great atmosphere, engaging the audience, bringing them together as one unit
	Watched a film/drama at cinema/other venue	33	Watching a film with others we all enjoyed at a drive-in movie surrounded by likeminded people all singing along in their own cars was really enjoyable and made me feel part of the experience
	Read as a past-time activity	28	Just the literature I read makes me feel I'm there with the people
	Been to an exhibition, museum, etc.	3	Just feel part of a bigger community visiting places of interest
	Played a musical instrument or sang	35	I play in the village Brass band. You feel like a team and a vital part of the community.
	Deliberately listened to recorded music	11	Part of a music scene bf is a DJ. He performs to an audience
	Listened to audio books or podcasts	23	I feel that podcasting is a very personal medium so I feel that it really makes me closer to people
	Attended a book club	5	Book clubs make me feel incredibly connected with those around me as due to the vibrance in the actual books makes me potentially feel interconnected with the book and my surroundings
	Been to an event connected with books/reading	7	Literature is my greatest love so when I'm at a literary event I feel like I'm in the sort of company that constitutes 'my tribe' in the world, the people with whom I have the most in common
	Done any form of crafts	6	Because I read, watch videos and do searches about it online and through social media, and through this I have seen that there are many people that like me like arts and crafts and also are similar to me in other aspects, which I didn't know before. Usually I don't really manage to connect in meaningful ways with others because I feel and am perceived as too different from the people I am around.
	Been to street art, public art displays, etc.	26	Street entertainment seems to create camaraderie between strangers, especially when it is unexpected and catches people by surprise. The

			public will often discuss what is going on with those around them, even if they have never met each other before
	Done photography, film, video making, etc.	11	Makes me feel like I've captured a part of them allowing them to become I little part of me
	Written as a past-time activity	3	Peaceful, everyone gets along with everyone. We are all one together
	Practised, rehearsed, or performed dance	5	Because I am part of a group striving to create something
	Done painting, drawing, printmaking, etc.	3	I feel like I am part of a group of people with shared interests
	Written or created music	2	As I feel part of team
	Practised, rehearsed, or performed a play, etc.	21	Rehearsing a play demands that you connect with your fellow performers. We aim to invoke emotion in our audience and so we have to connect with each other in order to feel those emotions ourselves. It's a deeply collaborative process, one which cannot achieve its full potential without connecting with each other. Even outside of rehearsals, you become a team - a family, even. Its a wonderful feeling of belonging
	Watched a dance performance live	11	Because you connect with all the people dancing and feeling so involved
	Been to a craft convention, show, or fair	3	Inclusive social outing
	'Other'	7	We practice together weekly (but sometimes on our own at home). We try to remember the steps, the pattern of the dance and the sound we are making with our clogs (made of wood and leather)…We have to work together as a team so that the sounds we make with our clogs ring out in unison, it makes us feel proud when we perform for ourselves and others and always puts a smile on my face
3.1.1 Enabling proximity to others	Been to a live musical performance	89	Because you are surrounded by other people
	Watched a theatre performance live	33	Theatre was small, stage was round and close to the audience. A sense of intimacy was evident
	Watched a film/drama at cinema/other venue	44	It's the only time I get a chance to spend time with family and friends so it's a great activity to do with people
	Read as a past-time activity	6	It enables to interact with people and their intimate experiences, even after they are passed away
	Been to an exhibition, museum, etc.	13	Being [in] a room full of people surrounded by art and just appreciating it

	Played a musical instrument or sang	4	Because it feels intimate to perform to people
	Deliberately listened to recorded music	27	Because when you are listening to music it brings back lifetime memories of who you was with and where you was the very first time you heard the songs being played
	Listened to audio books or podcasts	7	Podcasts offer the illusion of talking with friends
	Attended a book club	2	Because there is people around. I generally prefer to be alone
	Been to an event connected with books/reading	3	It gets people together
	Done any form of crafts	1	I'm with others
	Been to street art, public art displays, etc.	16	Good to be in company around other people
	Done photography, film, video making, etc.	11	It reminds me of outings and social events I have participated in with other people
	Written as a past-time activity	0	N/A
	Practised, rehearsed, or performed dance	2	Physical connection plus an element of synchronicity
	Done painting, drawing, printmaking, etc.	2	I think about people when I paint
	Written or created music	0	N/A
	Practised, rehearsed, or performed a play, etc.	2	Performance is always collaborative and brings people together to make and perform something. For example a theatre company putting on a show. It makes you feel a lot like a family and connected to each other and you feel sad when it comes to an end
	Watched a dance performance live	1	Good crowd
	Been to a craft convention, show, or fair	6	Just being around other people
	'Other'	0	N/A
3.2 Connecting through common interests	Been to a live musical performance	309	Everyone at the concert has the common interest of listening to that music
	Watched a theatre performance live	32	Being in the company of people with a common interest
	Watched a film/drama at cinema/other venue	39	Because I'm enjoying the same thing as the other people, shows we have similar interests

Read as a past-time activity	15	The connection when someone enjoys the same books that you do and when your child takes an interest in some of them and asks that you read them at bedtime, then starts asking questions about the content because they're taking it all in and want to understand it all better. It's all heartwarming, and the world suddenly feels a little less lonely
Been to an exhibition, museum, etc.	39	You get talking to other people with different ideas but same interests
Played a musical instrument or sang	15	Direct interaction with people who have the same interest
Deliberately listened to recorded music	36	People with similar tastes in music usually have similar interests in other things
Listened to audio books or podcasts	5	The podcasters are talking about something that interests both them and me. Listening to the same podcasters regularly makes me feel like I am getting to know them
Attended a book club	24	Because we connect over a love of books - spend time talking about books, sharing our own experiences and having time to relax with others
Been to an event connected with books/reading	28	Being around people with similar interests and views and discussing art feels like a wonderful, positive way to spend my time and in turn makes me feel connected to them
Done any form of crafts	9	Being able to share and ideas with people passionate about the same things as me
Been to street art, public art displays, etc.	21	I enjoy visiting galleries and historic houses. This brings me closer to my family who also enjoy these things, particularly my daughters as we share a similar interest in fine art and my young grandsons who are both very interested in history
Done photography, film, video making, etc.	10	I feel connected to my partner as we enjoy doing this together
Written as a past-time activity	2	Because the (informal) 'newsletter' I write is written for a specific group of individuals and the theme is a common interest we all share
Practised, rehearsed, or performed dance	8	The action of coming together with others who's only connection is their enjoyment for acting singing and dancing. Then creating something with those new friends
Done painting, drawing, printmaking, etc.	6	We all share the love for arts

	Written or created music	4	You connect with people through your love of music. In my opinion, the purest form of understanding and getting to know people
	Practised, rehearsed, or performed a play, etc.	4	Sharing a common interest. Going out to take part in activities. Social life derived from participation in such activities. Providing entertainment for others to enjoy
	Watched a dance performance live	6	We all love the same dance it becomes a new family
	Been to a craft convention, show, or fair	12	I feel that when I'm at a craft fair other people are as interested as me therefore we have a connection
	'Other'	6	Because it's all people united in one common interest
3.3 Connecting with likeminded people	Been to a live musical performance	75	Lots is like-minded people getting together to listen to music they all share a passion for
	Watched a theatre performance live	18	I went to the theatre to see a drag queen. Everyone in the audience was accepting and like-minded which made me feel more connected to others. Additionally the drag queen I went to see was very involved with the audience by interacting with them in conversation and physically. This made me feel more connected to them than if they performed without interaction. They also pretended to take away the "wall" between the audience and stage
	Watched a film/drama at cinema/other venue	5	Because I go with family or friends and I am surrounded by likeminded people who have gone to see the same movie
	Read as a past-time activity	2	I think how someone connects with a story/character says a lot about them and I like to engage with people who feel the same way towards a certain story/character as I do
	Been to an exhibition, museum, etc.	11	Being with like-minded people who enjoy visiting museums etc.
	Played a musical instrument or sang	7	Being part of a community choir makes you feel you belong to a group of likeminded people who are enjoy the sense of togetherness and singing for pleasure
	Deliberately listened to recorded music	3	I have met likeminded people through this in the past
	Listened to audio books or podcasts	3	It helps me identify with likeminded people
	Attended a book club	13	Takes something that gives me pleasure as an individual pastime and enables me to share it with likeminded people. It makes me engage with

		the books discussed in a different way as enjoy it not just for it's own sake but for how I can get more out of it from an alternative perspective
Been to an event connected with books/reading	9	Because you get to meet likeminded people and talk to them
Done any form of crafts	9	Because I read, watch videos and do searches about it online and through social media, and through this I have seen that there are many people that like me like arts and crafts and also are similar to me in other aspects, which I didn't know before. Usually I don't really manage to connect in meaningful ways with others because I feel and am perceived as too different from the people I am around. So even though I have not really personally connected to the arts and crafts people, I feel as part of the community and I have hope that when I feel ready I will be able to join said community in a more active way
Been to street art, public art displays, etc.	5	Can enjoy with likeminded people
Done photography, film, video making, etc.	7	I love doing night sky photography and recently purchased a Canon camera. I meet up with other enthusiasts to photograph the moon, Orion s belt and the stars. And this activity does allow you to get connected with other likeminded people
Written as a past-time activity	0	N/A
Practised, rehearsed, or performed dance	2	I attend Line dancing with a great bunch of likeminded people
Done painting, drawing, printmaking, etc.	5	My work has been appreciated by likeminded people who look forward to viewing more work in the future
Written or created music	0	N/A
Practised, rehearsed, or performed a play, etc.	2	Yes it’s great because i get to be surrounded by likeminded people
Watched a dance performance live	2	Enjoy being with like-minded people
Been to a craft convention, show, or fair	8	Meeting likeminded people
‘Other’	4	Special exhibitions at museums - which are advertised, and therefore attract like-minded people

Overarching theme 4 Facilitating collective understanding

Sub-theme/sub-sub-theme	*Arts activity*	*Frequency*	*Indicative evidence*
4.1 Learning from and with others	Been to a live musical performance	3	Share the experience together, learn about new music
	Watched a theatre performance live	1	Watching other performers on the stage sets the bar for where you're reaching so when we showcase our talents to the world we are not only blasting above our own standards (when doing it with passion) we are hopefully inspiring and showing others what they are capable of
	Watched a film/drama at cinema/other venue	5	Usually go with family so after event we always go for a meal and chat about the film. Makes it proper family time.
			The recent memorable example was when we went to see darkest hour and I explained to my 18 year old son about the history of those times to put the film into perspective
	Read as a past-time activity	16	You're taken into people's lives and learn from them
	Been to an exhibition, museum, etc.	8	I get to enjoy it with my young son while educating him about the world
	Played a musical instrument or sang	7	I sing with a community choir so it is very much about connecting with each other through listening, learning together and growing together. Through a recent bereavement it has been sometimes the only way I can relate to other people and it has given me the strength to carry on with my life as best I can
	Deliberately listened to recorded music	1	Shared knowledge
	Listened to audio books or podcasts	7	It's a topic where even if they don't know much about you can still talk and teach them in a way of the topic
	Attended a book club	3	Talking to other people Helps with communication skills and confidence Therefore making your feel more connected
	Been to an event connected with books/reading	3	Share my knowledge
	Done any form of crafts	22	My sister and I went to a pottery class last month, and as well as earning new techniques together, we managed to catch up, give each other advice, and take a keepsake of our special day
	Been to street art, public art displays, etc.	2	Sharing in learning about how monuments art and architecture fit in to history and bring it to life together

	Done photography, film, video making, etc.	7	Because I work alongside others and can throw ideas and solutions at each other. They make me better and I make them better. We complement each other
	Written as a past-time activity	5	Learn more find out more
	Practised, rehearsed, or performed dance	8	As you learn together you develop a bond
	Done painting, drawing, printmaking, etc.	6	Because you have to work with others and share ideas
	Written or created music	4	I have to speak to others for advice
	Practised, rehearsed, or performed a play, etc.	3	Joint production gives support from a group and a mutual sense of achievement
	Watched a dance performance live	0	N/A
	Been to a craft convention, show, or fair	4	Being able to share experiences and learn new skills
	'Other'	5	I get great feedback and it makes me feel part of something. I feel like I'm helping others and I feel helped myself
4.2 Relating to others	Been to a live musical performance	4	Relate to others more readily
	Watched a theatre performance live	8	Because there are real people representing life situations that I can relate to and admire
	Watched a film/drama at cinema/other venue	1	Some events replicate certain phases of my life
	Read as a past-time activity	10	It stops you feeling so alone, when someone else can describe a situation the same way you experience it
	Been to an exhibition, museum, etc.	7	I love History for this I would take my grandkids and family to museums and historical sites and try and put ourselves in the past with the people
	Played a musical instrument or sang	3	Other people into the same things as me make me feel at home, more than being at home does. I like relating to people
	Deliberately listened to recorded music	7	You can connect with people more through music. The lyrics can relate to you and your life
	Listened to audio books or podcasts	10	Relating to their stories. Makes me feel like I'm not alone. Sharing stories. Help feel normal
	Attended a book club	1	We share thoughts on the book and relate its content to our own lives

	Been to an event connected with books/reading	3	I suppose it's usually when the speaker or author is someone I admire or respect. Often their experiences and what they write about reflect my own experiences and I feel less alone
	Done any form of crafts	1	It's a knitting group with a wide age range. The founder set it up as a group to help with mental health issues. It's sociable, inspirational and makes what could be a solitary pastime into one to be shared
	Been to street art, public art displays, etc.	1	It is the shared sense of witnessing something. It gives it a certain sense of permanence, and timelessness. Going to see something as evocative and inspiring as the reclining Buddha in Bangkok makes you think of all the other people that have stood where you are. It makes you contemplate how they felt. It gives you a far greater depth of perception and to my mind increases your human consciousness.
	Done photography, film, video making, etc.	3	Capturing people on film makes you reflect on yourself and how you relate to others
	Written as a past-time activity	2	Relate to others with similar interests
	Practised, rehearsed, or performed dance	0	N/A
	Done painting, drawing, printmaking, etc.	2	It releases stress which is useful and other people can relate to it so that is why I feel connected to other people
	Written or created music	0	N/A
	Practised, rehearsed, or performed a play, etc.	1	A group for people with mental health problems
	Watched a dance performance live	1	Well I've danced since I was little, it was always a passion and i had to give it up unfortunately so seeing all these extremely talented dancers put me at ease with everything
	Been to a craft convention, show, or fair	0	N/A
	'Other'	1	Because the people that go there have mental health difficulties like me
4.3 Learning about others	Been to a live musical performance	0	N/A
	Watched a theatre performance live	2	Because it makes me think of the history of the play/ performance and I wonder what life was like for them
	Watched a film/drama at cinema/other venue	3	It's a good way to understand our world and other people. Humans are together and they can share a culture and traditions

Read as a past-time activity	28	It opens up new worlds and new ideas. It helps me to understand the lives and experiences of other people
Been to an exhibition, museum, etc.	18	Being outside and exploring with someone particularly if it has a historical interest where you can feel connected to the people before us
Played a musical instrument or sang	3	I run a community choir, it helps us all learn about each other and to see everyone progress with their confidence and participation really is the best thing. It has really helped me get to know my local area and the people living here
Deliberately listened to recorded music	4	Music is a powerful tool for understanding emotions and intentions and beliefs of the artist performing it. It says more than just regular words and sharing that music with peers and getting their take on it does the same
Listened to audio books or podcasts	10	The podcasters are talking about something that interests both them and me. Listening to the same podcasters regularly makes me feel like I am getting to know them
Attended a book club	0	N/A
Been to an event connected with books/reading	3	You learn more about the authors or the artwork that your seeing. I feel that you connect more on a personal level
Done any form of crafts	1	Brings out each individuals creative side
Been to street art, public art displays, etc.	6	You meet people from all walks of life. Socialising and learning new things
Done photography, film, video making, etc.	3	I feel I connect with other people through photography. I get to spend time with people. Listen to their stories. I try to capture their emotions, what they've been through and what they are currently going through
Written as a past-time activity	2	because I feel that I have to cater to other peoples interest and so ensure to research this in depth 1st
Practised, rehearsed, or performed dance	1	Because it gives me the ability to understand the people I am around. Doing something that connects us all
Done painting, drawing, printmaking, etc.	0	N/A
Written or created music	1	Supporting others in expressing themselves

	Practised, rehearsed, or performed a play, etc.	0	N/A
	Watched a dance performance live	1	Because it gives me the ability to understand the people I am around. Doing something that connects us all
	Been to a craft convention, show, or fair	1	To realise what other people can make or do
	'Other'	0	N/A
4.4 Connecting with different people and cultures	Been to a live musical performance	19	They usually involve people from various backgrounds, of different ages but all with a love of music in common so instill a sense of common ground, no matter the otherwise diversity of the attendees
	Watched a theatre performance live	4	So many people of every background all coming together to watch and enjoy something amazing
	Watched a film/drama at cinema/other venue	1	It's a good way to understand our world and other people. Humans are together and they can share a culture and traditions
	Read as a past-time activity	2	Because I learn new thing about the world, the people and different cultures and ideologies
	Been to an exhibition, museum, etc.	4	You get such a variety of people at art venues of museums, all sharing an experience which makes me feel like part of the wider world
	Played a musical instrument or sang	3	Music brings people together. Dont matter where are you from, or who you are, music is equal. A good company, a good music, outside in the nature, playing on guitar, every music is emotions. Music is really important, they talk louder than any words what people can say
	Deliberately listened to recorded music	1	Because a shared love of music, sometimes a specific type, shows that regardless of age or other interests, there is a common bond that can be explored…[It] may make someone realize that people who may seem very different, may not be so different after all
	Listened to audio books or podcasts	1	Reading gives you many ways of imagination around different people
	Attended a book club	3	Because I can get to know more about different types of people
	Been to an event connected with books/reading	2	It brings different people from different countries and nationalities, performers
	Done any form of crafts	2	When I am doing this, particularly in public, it attracts attention and many people from all sorts of backgrounds and countries come up to me and

			speak to me. They also share with me the sorts of things they do and are interested in
	Been to street art, public art displays, etc.	6	Cause anyone can come there you meet a lot of nice and interesting people
	Done photography, film, video making, etc.	0	N/A
	Written as a past-time activity	1	Share cultures and way of life
	Practised, rehearsed, or performed dance	1	Same level of expertise, like minded, various age range, good atmosphere
	Done painting, drawing, printmaking, etc.	0	N/A
	Written or created music	0	N/A
	Practised, rehearsed, or performed a play, etc.	0	N/A
	Watched a dance performance live	1	I like to see people perform and from different cultures to learn more
	Been to a craft convention, show, or fair	0	N/A
	'Other'	2	Fun activity for all ages in the community
4.5 Connecting with different viewpoints	Been to a live musical performance	3	Music brings everyone together regardless of any religious, political or ethnic beliefs
	Watched a theatre performance live	0	N/A
	Watched a film/drama at cinema/other venue	0	N/A
	Read as a past-time activity	2	Helps me see the world in a new and different way, regarding culture and intellectual pursuits
	Been to an exhibition, museum, etc.	10	Because we are looking at the same thing but see different things but can discuss it
	Played a musical instrument or sang	1	Because everyone can sing, not well or intune but none the less everyone can sing. Everyone can understand a song but not everyone can understand it's true meaning. But songs have many meanings so it is fine if you don't get what the writer or singer means
	Deliberately listened to recorded music	2	Imagining other peoples interpretations
	Listened to audio books or podcasts	1	Hearing other points of view

Attended a book club	6	It gives you a chance to see other perspectives and hear new ideas, seeking those things out is one of the most human things you can do
Been to an event connected with books/reading	1	Understand people's views interpretation
Done any form of crafts	0	N/A
Been to street art, public art displays, etc.	0	N/A
Done photography, film, video making, etc.	1	By seeing things from different points of view, prompting conversation
Written as a past-time activity	0	N/A
Practised, rehearsed, or performed dance	0	N/A
Done painting, drawing, printmaking, etc.	1	I find drawing and painting a relaxing and rewarding experience whether alone or as member of a group. It is amazing that a number of people can draw or paint a specific scene or object but their interpretation will appear different in almost every case and can become the topic of in depth conversation
Written or created music	0	N/A
Practised, rehearsed, or performed a play, etc.	0	N/A
Watched a dance performance live	0	N/A
Been to a craft convention, show, or fair	1	This make me think about how other people interpret and see things. It makes me see how we can see things differently. Like an abstract sculpture can be seen to appear very different to me than the person who created it, however I feel connected as we can both see something united
'Other'	0	N/A
